# Transcutaneous electrical nerve stimulation as an adjuvant treatment for thoracolumbar acute hyperesthesia in chondrodystrophic dogs: a prospective blinded controlled clinical study

**DOI:** 10.3389/fpain.2025.1496607

**Published:** 2025-03-21

**Authors:** Débora Gouveia, Ana Cardoso, Carla Carvalho, Marina Moisés, André Coelho, Maria Manuel Balça, Rui Alvites, Ana Colette Maurício, António Ferreira, Ângela Martins

**Affiliations:** ^1^ Arrábida Animal Rehabilitation Center, Arrábida Veterinary Hospital, Setubal, Portugal; ^2^ Superior School of Health, Protection and Animal Welfare, Polytechnic Institute of Lusophony, Lisboa, Portugal; ^3^ Faculty of Veterinary Medicine, Lusófona University, Lisboa, Portugal; ^4^ Departamento de Clínicas Veterinárias, Instituto de Ciências Biomédicas de Abel Salazar (ICBAS), Universidade do Porto (UP), Rua de Jorge Viterbo Ferreira, Porto, Portugal; ^5^ Centro de Estudos de Ciência Animal (CECA), Instituto de Ciências, Tecnologias e Agroambiente da Universidade do Porto (ICETA), Porto, Portugal; ^6^ Associate Laboratory for Animal and Veterinary Science (AL4AnimalS), Lisboa, Portugal; ^7^ Instituto Universitário de Ciências da Saúde (CESPU), Gandra, Portugal; ^8^ Faculty of Veterinary Medicine, University of Lisbon, Lisboa, Portugal; ^9^ CIISA - Centro Interdisciplinar-Investigação em Saúde Animal, Faculdade de Medicina Veterinária, Av. Universi Dade Técnica de Lisboa, Lisboa, Portugal

**Keywords:** TENS, neuropathic pain, IVDD, paraspinal hyperesthesia, DIVAS

## Abstract

**Introduction:**

Acute paraspinal hyperesthesia in dogs can result in a combination of nociceptive and neuropathic pain, often requiring pharmacological intervention. However, non-pharmacologic approaches, such as two-channel transcutaneous electrical nerve stimulation (TENS), may also be beneficial. Evidence from human medicine suggests that conventional TENS reduces pain scores and potentially decreases the need for analgesic medication. This study aimed to evaluate the efficacy of TENS as an adjunctive treatment for thoracolumbar paraspinal hyperesthesia in dogs.

**Methods:**

This prospective, blinded, controlled cohort study was conducted in a clinical setting. Dogs diagnosed with paraspinal hyperesthesia, classified as grade 4 or 5 on the modified Frankel scale (MFS) and with a dynamic interactive visual analog scale (DIVAS) score ≥14, were included. The subjects were randomized into two groups: the study group (SG), which received standard pharmacological protocol (PSP) plus TENS, and the control group (CG), which received PSP only. Observers blinded to treatment allocation scored video recordings of the dogs. Assessments were performed every 24 hours from T0 (admission) to T8, with evaluations in SG occurring 5 minutes before each TENS session.

**Results:**

A total of 818 dogs were enrolled, with 605 (74%) in the SG and 213 (26%) in the CG. In the first 48 hours, SG demonstrated a faster reduction in muscle tone compared to CG. While all dogs transitioned from a hyperesthetic to a non-painful state, SG showed a significantly faster recovery from T2 (48 h) to T4. A significant difference was observed between groups in DIVAS scores (*p* < 0.001). Additionally, SG had a shorter mean hospital stay (2.14 days) compared to CG, which required twice as long (*p* < 0.001).

**Discussion:**

These findings suggest that TENS may be an effective adjunctive therapy for managing acute thoracolumbar hyperesthesia in dogs, promoting early recovery by reducing pain, medication dependency, and hospitalization duration. However, the study's reliance on subjective assessments presents a limitation, potentially introducing bias. Further research with objective outcome measures is necessary to validate these findings and optimize the integration of TENS in veterinary pain management protocols.

## 1 Introduction

Transcutaneous electrical nerve stimulation (TENS) is a rehabilitation method traditionally used to treat pain in both veterinary and human medicine (1). The American Physical Therapy Association (2) defines TENS as an application of electrical stimulation to the skin for the treatment of pain. This neuromodulation technique is indicated as an adjunctive therapy to relieve pain sensations, muscle tension, and spasms and reduces the need for analgesics (3, 4). Electrical stimulation through surface electrodes can be easily performed and has shown positive and effective results in controlling lower limb musculature spasticity in humans (5, 6), with long-term benefits when applied in multiple sessions (5). TENS is usually performed at either high or low frequencies (7), and several studies describe the use of high frequencies above 100 Hz (8–10). In contrast, low frequencies are defined as ∼10 Hz or less (9, 11, 12). In 1965, Melzack and Wall introduced the “pain gate” theory, which assumes large diameter afferent fibers (A*β*) that inhibit central nociceptive transmission, leading to a decrease in pain perception. This is one of the possible mechanisms of action behind TENS therapy (13). The effects of TENS via the spinal segments are reducing inflammation by sensitizing neurons of the dorsal horn (12); altering levels of neurotransmitters, such as gamma-aminobutyric acid (GABA) and glycine; inhibiting nociceptive traffic (14, 15); and modulating the activity of cells in the spinal cord that support and surround neurons (glial cells) (16). The pathways by which TENS may promote analgesia at the spinal level (7, 17). In addition, there appears to be some mediation through *μ*- and *γ*-opioid receptors as well as activation of serotonin receptors (18, 19). The most common cause of spinal cord injury (SCI) in dogs is intervertebral disc disease (IVDD) with possible secondary compressive myelopathy (20, 21), which mostly occurs in dogs of chondrodystrophic breeds, such as French bulldogs and dachshunds (22). Thoracolumbar IVDD is reported with a prevalence of 66%–87% (21) and a higher risk in the intervertebral spaces T12-T13 and T13-L1 (23–26), possibly leading to various clinical manifestations (22, 27). This acute myelopathy is a major cause of paraspinal hyperesthesia (28). The aim of this prospective, blinded, controlled observational clinical cohort study was to evaluate the effects of TENS in dogs with acute thoracolumbar hyperesthesia. We hypothesized that TENS could have a role as an effective and safe adjunct therapy to the standard pharmacological protocol for paraspinal hyperesthesia in the clinical setting.

## 2 Materials and methods

This prospective, observational, blinded, controlled, clinical cohort study was based on data collection between January 2019 and March 2024 in two rehabilitation centers of the Arrábida Veterinary Hospital (HVA, Portugal): Centro de Reabilitação Animal da Arrábida (CRAA) and Centro de Reabilitação e Regeneração Animal de Lisboa (CR2AL). The study was approved by the Ethics Committee of the Faculty of Veterinary Medicine of Lusófona, and all owners signed an informed consent form (No. 18-2024).

### 2.1 Population presentation

The study population included all dogs with paraspinal hyperesthesia in the thoracolumbar region with a neurologic status grade of 4 or 5 on the modified Frankel scale (MFS), i.e., dogs with ataxic or normal gait and unremarkable orthopedic examination. Participants were selected if they were chondrodystrophic, ≤7 years old, and weighed ≤25 kg, regardless of their sex. In addition, dogs were only selected if they had a dynamic interactive visual analog scale (DIVAS) classification ≥14. Dogs with a history of seizures, pregnancy, cardiac instability, discospondylitis, and dermatological problems were excluded from the study. In addition, all dogs that had paraspinal hyperesthesia but had already undergone surgery were excluded. At admission (T0), all dogs (*n* = 818) were observed and filmed (Canon EOS Rebel T6 1300 D camera) to assess paraspinal hyperesthesia, paraspinal muscle tone, and muscle tone of the limbs and abdomen. All were assessed using the DIVAS, which is a subjective, non-validated numerical rating scale that is intuitive for pain assessment (29, 30) and allows hyperesthesia in the thoracolumbar region of the vertebral column to be assessed by palpation. Palpation itself is considered the most widely used clinical method for detecting pain in dogs (31).

### 2.2 Study design

The dogs were referred from the HVA to the rehabilitation centers of CRAA and CR2AL and divided into two groups depending on whether the owner agreed to the TENS treatment or not. This convenience recruitment of dogs resulted in the study group (SG) with dogs that underwent the two-channel TENS and the standard pharmacological protocol (PSP), while the control group (CG) consisted of dogs that underwent only the PSP. Both had the same approach to medication and environment and did not perform any other rehabilitation interventions. During hospitalization, they performed cage rest and PSP: meloxicam (0.1 mg/kg SC) SID for 3 days; paracetamol (10 mg/kg IV) TID, depending on paraspinal pain for three to 7 days; gabapentin (5 mg/kg per os) TID; and methadone (0.1 mg/kg IV) BID. These medications were discontinued depending on the individual patient's recovery. In addition, all performed fluid therapy with Ringer's lactate (3–5 mg/kg) and gastrointestinal nutrition (EN Gastrointestinal Diet, Purina Pro Plan) for the first 12 h. Cage rest consisted of a cage with a non-slip floor that provided enough space for the dog to stand up and take a step or two, but in a conditioned environment that restricted movement.

**Figure 1:**
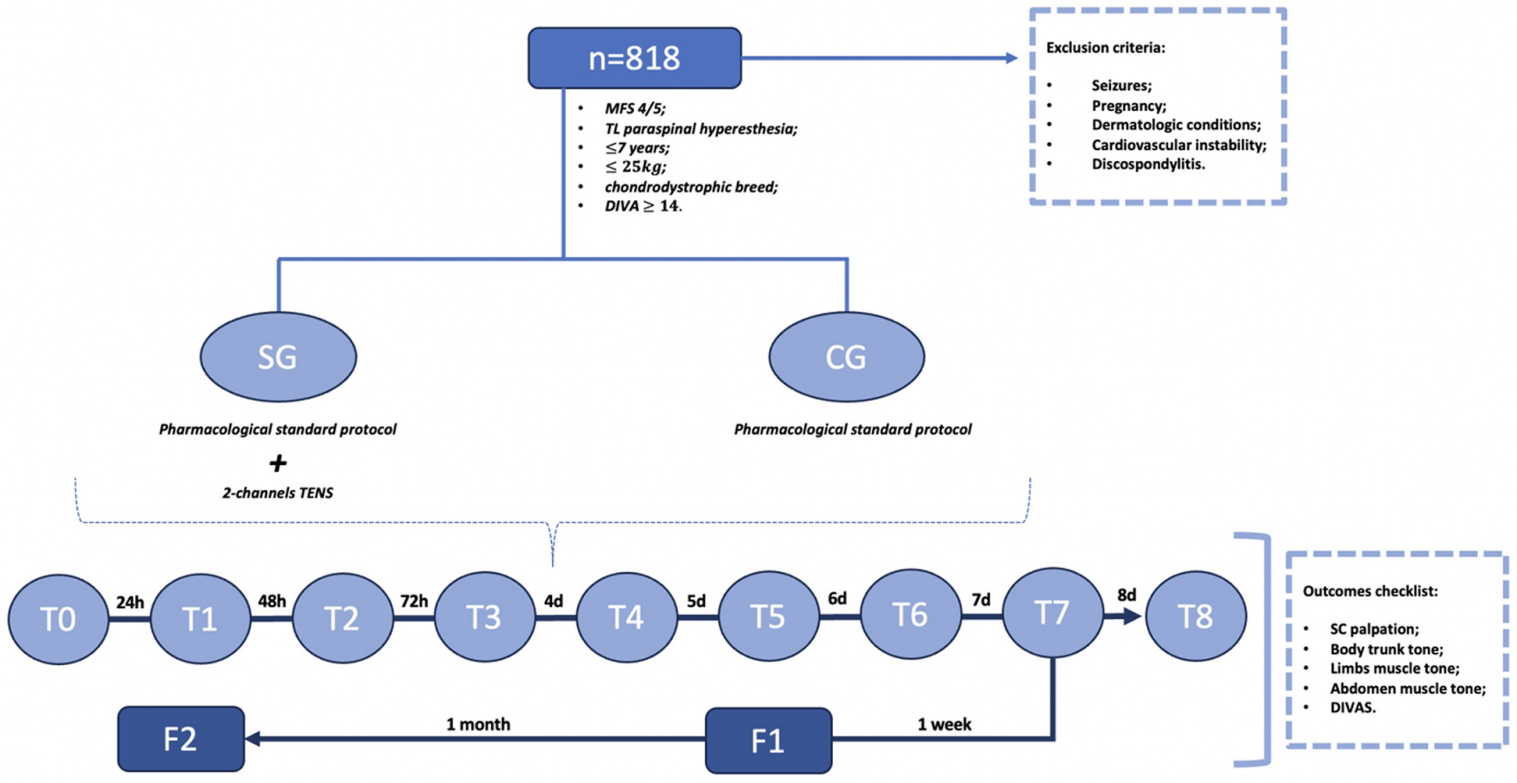
Flow diagram of the clinical study. MFS, modified Frankel scale; TL, thoracolumbar; DIVAS, dynamic interactive visual analog scale; SG, study group; CG, control group; TENS, transcutaneous electrical nerve stimulation; T, time; F, follow-up; SC, spinal cord.

The dogs in both groups were examined on admission (T0); after 24 h (T1), 48 h (T2), 72 h (T3), 4 days (T4), 5 days (T5), 6 days (T6), 7 days (T7), and 8 days (T8); and at follow-up examinations after 1 week (F1) and 1 month (F2) (Figure 1). Since it is a study in a clinical setting of a hospital, the 818 dogs were not admitted by the same veterinarian. Although, when admitted in HVA, dogs were examined by different veterinarians with expertise in the field, it was the same veterinarian that carried out the examinations between T1 and T8 and follow-up examinations (F1 and F2), performed in the same controlled environment of the rehabilitation centers.

Two blinded observers, both certified canine rehabilitation veterinarians (CCRP) and a diplomate of the European College of Veterinary Sports Medicine and Rehabilitation (Dipl. ECVSMR), were responsible for the video recording analysis of the results at all time points. All rehabilitation veterinarians and nurses participating in this multidisciplinary treatment were also blinded to the results of the video recording evaluation. The interobserver disagreement was obtained by comparing the totality of classifications from the checklist outcomes from the two observers and identifying the ones that were not agreeing.

### 2.3 Outcomes

This study is established on a subjective outcome checklist, which is based on the neurorehabilitation examination, but has not been validated. The reaction/behavior of each dog was recorded for all the parameters mentioned above, for further analysis. Each parameter is described in detail as follows.

#### 2.3.1 Palpation of the spinal cord to evaluate paraspinal hyperesthesia

**Figure 2:**
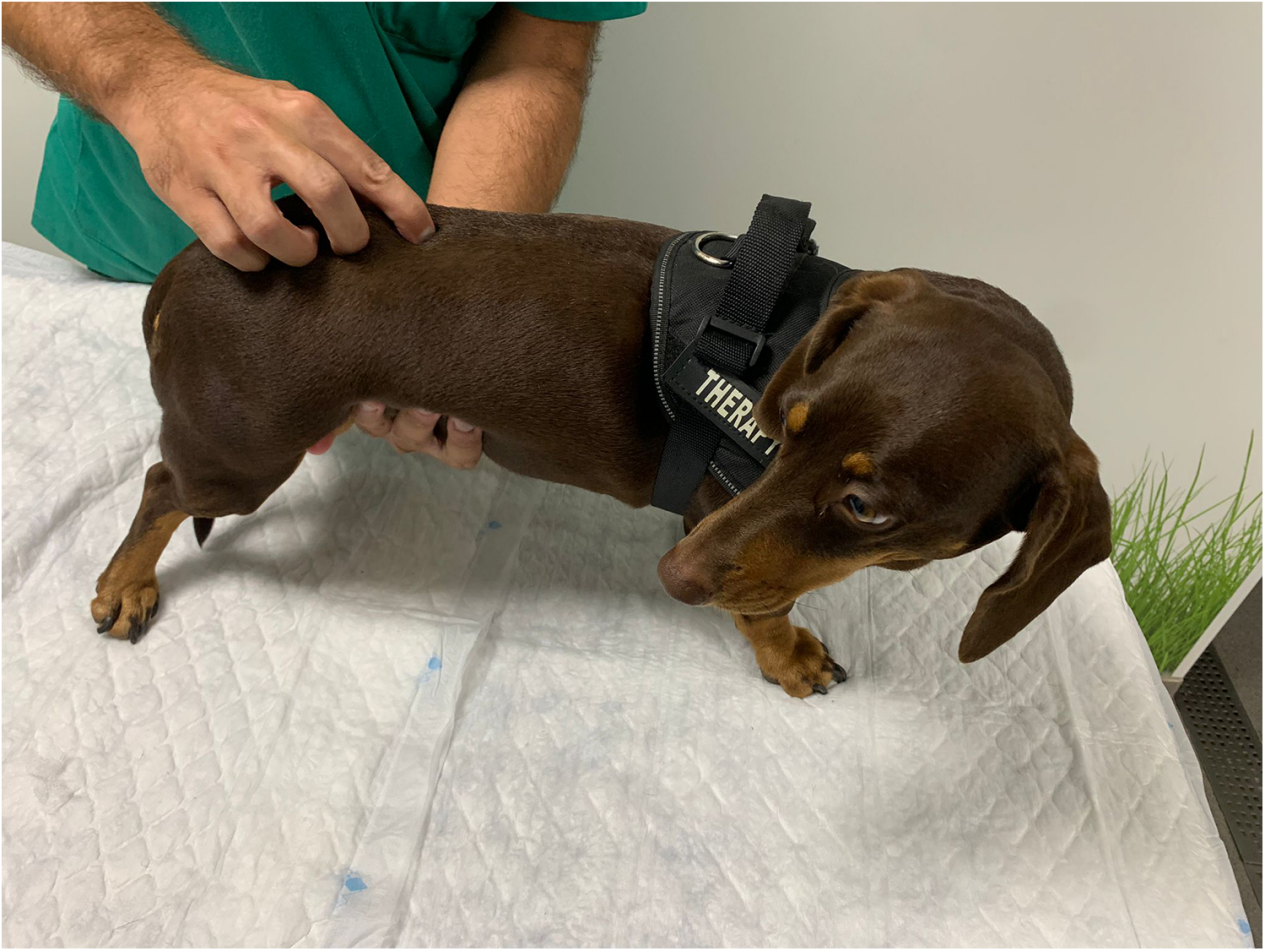
Paraspinal hyperesthesia assessment through vertebral column palpation.

Paraspinal hyperesthesia was assessed using the spinal palpation technique and categorized as present or absent. This technique involved applying light pressure to the spine, placing a hand on the abdominal muscles, and palpating the entire supraspinal ligament between each spinous process and between each intervertebral space to activate the nociceptors in the peripheral *annulus fibrosus*, starting in the interscapular region and moving caudally to the lumbosacral region (32–34) (Figure 2).

#### 2.3.2 Paraspinal muscle tone evaluation

**Figure 3:**
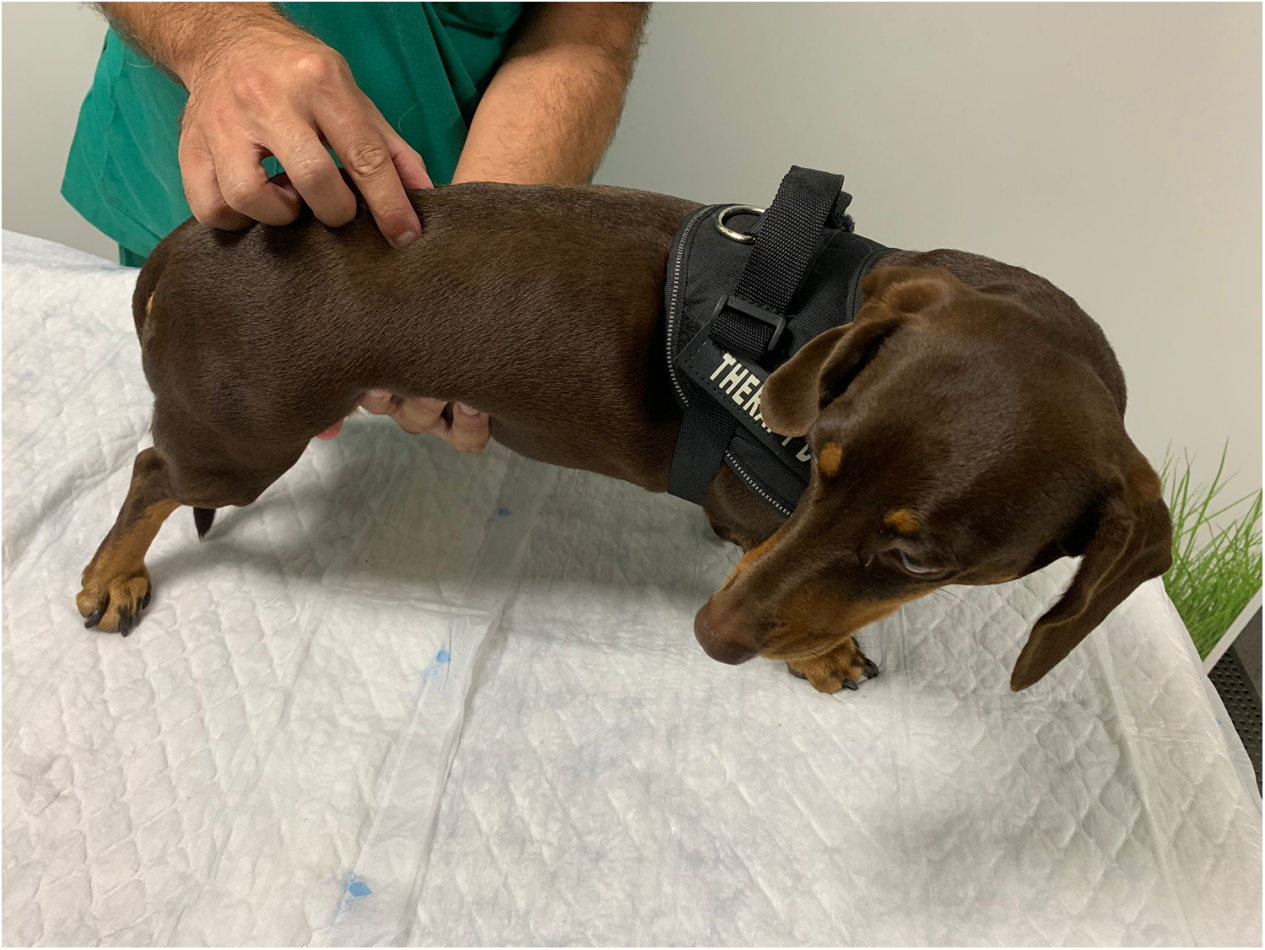
Evaluation of the paraspinal muscle tone by paraspinal muscle palpation.

The assessment of paraspinal muscle tone was performed with the rehabilitator behind the patient who was in a standing position. The paraspinal muscles were palpated along the entire vertebral column from cranial to caudal, always paying attention to attention to several parameters, such as postural changes (e.g., kyphosis, lordosis, scoliosis) (Figure 3). This assessment parameter was classified as normal, increased, or decreased. It was considered normal when there were no reaction or posture alterations of the dog upon muscle tone palpation.

#### 2.3.3 Limb muscle tone evaluation

**Figure 4:**
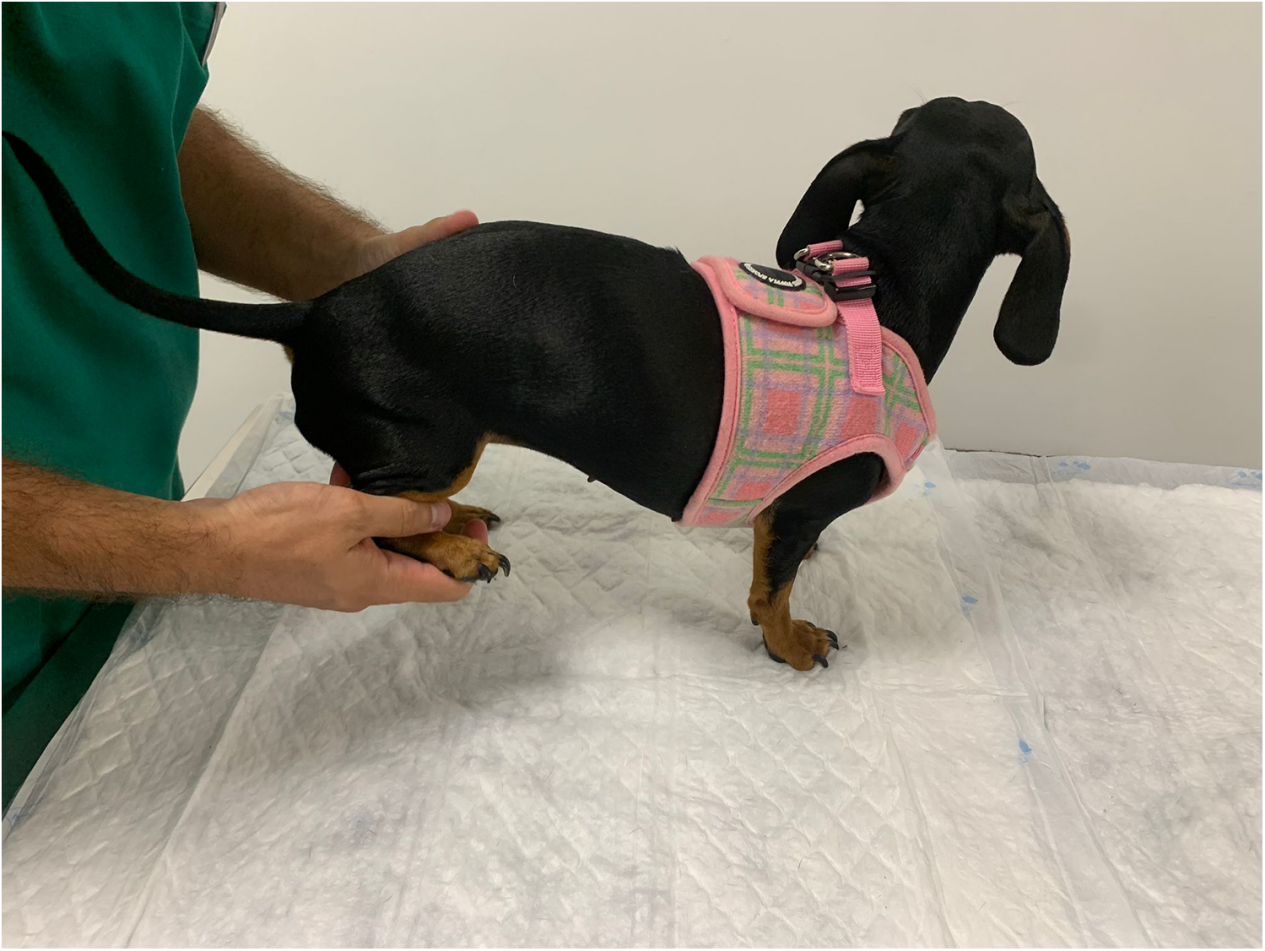
Palpation of the muscle tone and flexion of the three hindlimb joints.

Palpation of the muscle tone of the hind and forelimbs was performed with the rehabilitator behind the patient who was in a standing position. The extensor muscle group was assessed first, followed by the flexor muscle group, assessing the retraction reflex and flexion of the three joints at the same time (Figure 4), allowing focal muscle atrophy and possible orthopedic changes to be detected. This assessment parameter was classified as normal, increased, or decreased. It was considered normal when there was no muscle rigidity or spasticity, combined with a normal retraction reflex and without a stiffness reaction to the flexion of the joints.

#### 2.3.4 Abdominal muscle tone evaluation

**Figure 5:**
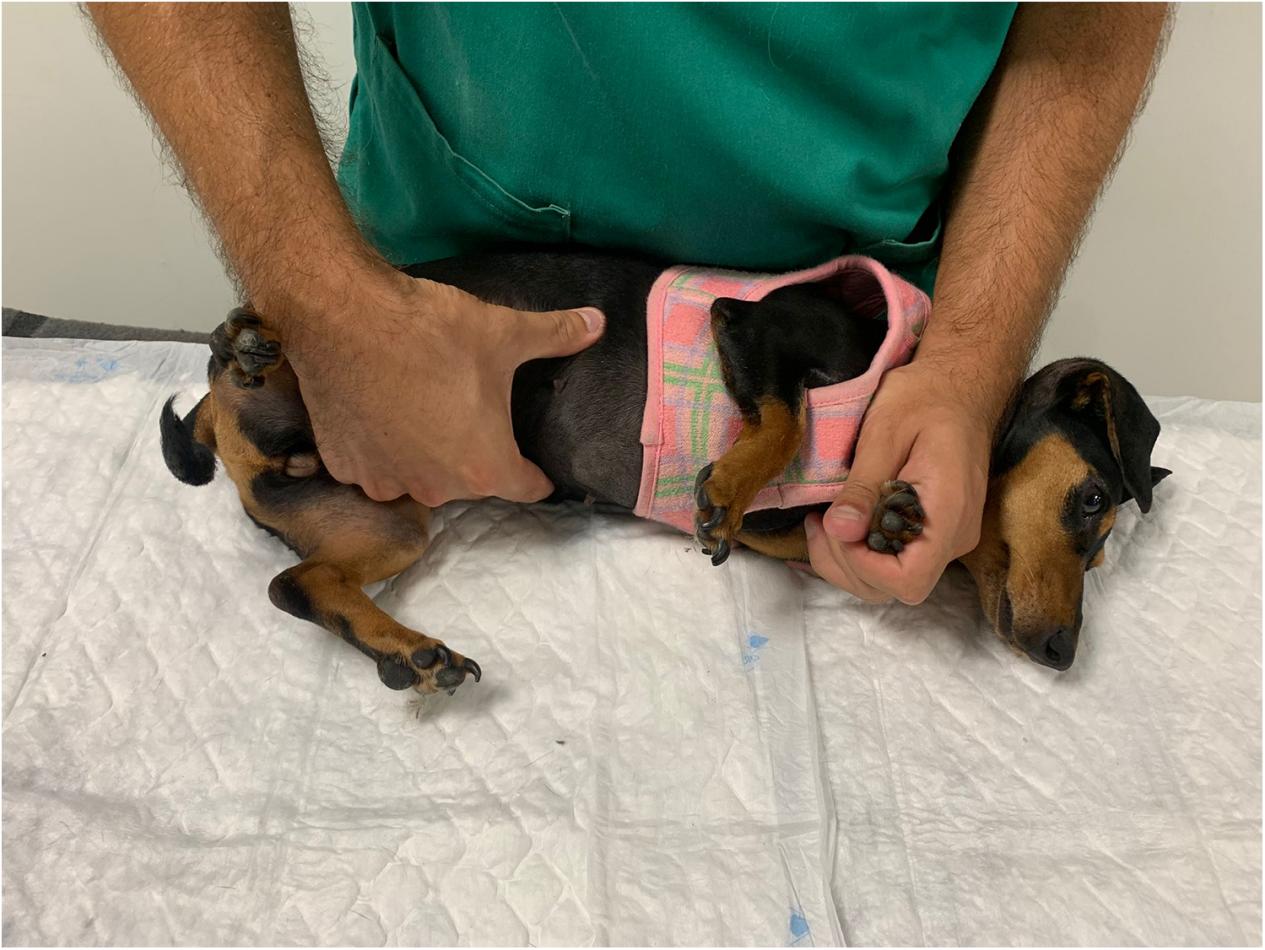
Palpation of the abdominal muscle tone in lateral recumbency.

In the same position, the rehabilitator evaluates the abdominal wall with the dog in an active standing position or in a lateral position (Figure 5), avoiding any possible discomfort to the patient. This assessment parameter was classified as normal, increased, or decreased. It was considered normal when there was no reaction of abdominal contraction upon palpation, regardless of the standing or lateral position.

#### 2.3.5 Dynamic interactive visual analog scale (DIVAS)

The DIVAS, a subjective tool and a non-validated numerical scale (0–27), was performed considering the following classifications: 1–5 (mild pain), 6–13 (moderate pain), 14–21 (severe pain), and 21–27 (unbearable pain). DIVAS is based on several assessment points, such as the physiological parameters, response to palpation, and possible protective reactions (e.g., head turning, aggressive behavior, and attempt to run away), observation of activity, posture, vocalization, and state of mind (Table 1) (35). This is a scale that can be easily applied by the rehab veterinarian. It provides a wealth of information about the patient and reduces the possibility of bias.

**Table 1:** Dynamic interactive visual analog scale (DIVAS) (35).

Physiological Parameters	a) Physiological data within the reference range	**0**
	b) Dilated pupils	**2**
	Increase in heart rate (relative to baseline)	
	>20%	**1**
	>50%	**2**
	>100%	**3**
	Increase in respiratory rate relative to baseline	
	>20%	**1**
	>50%	**2**
	>100%	**3**
	Hyperthermia	**1**
	Salivation	**2**
Response to palpation	No behavioral changes	**0**
	Protective reactions*/when touched	**2**
	Protective reactions*/before touching it is protected	**3**
	* Movements of the head to the affected area, licking, biting, scratching the wound, tensing muscles, and protective posturing	
Activity	a) At rest: sleeping	**0**
	b) At rest: semi-conscious	**0**
	c) At rest: awake	**1**
	d) Eating	**0**
	e) Restless (walk constantly, gets up and back to bed)	**2**
	f) Sudden and frequent movements (wallowing and hitting)	**3**
State of mind	Submissive	**0**
	Anxious	**1**
	Fearful	**2**
	Aggressive	**3**
Posture	Protects the affected area (fetal position)	**2**
	Lateral decubitus	**0**
	Sternal decubitus	**1**
	Choose one:	
	Sitting or standing	**2**
	Moving	**1**
	Abnormal posture	**2**
Vocalization	a) Does not vocalize	**0**
	b) Vocalizes when touched	**2**
	c) Intermittent vocalization	**2**
	d) Continuous vocalization	**3**

The numerical values, in bold, correspond to the classification that is attributed to each parameter. Considering the sum of the classification attributed to each parameter, the results were interpreted as follows: description of pain-1–5 = mild pain; 6–13 = moderate pain; 14–21 = severe pain; 21–27 = unbearable pain.

### 2.4 Interventions

#### 2.4.1 Two-channel transcutaneous electrical nerve stimulation

**Figure 6:**
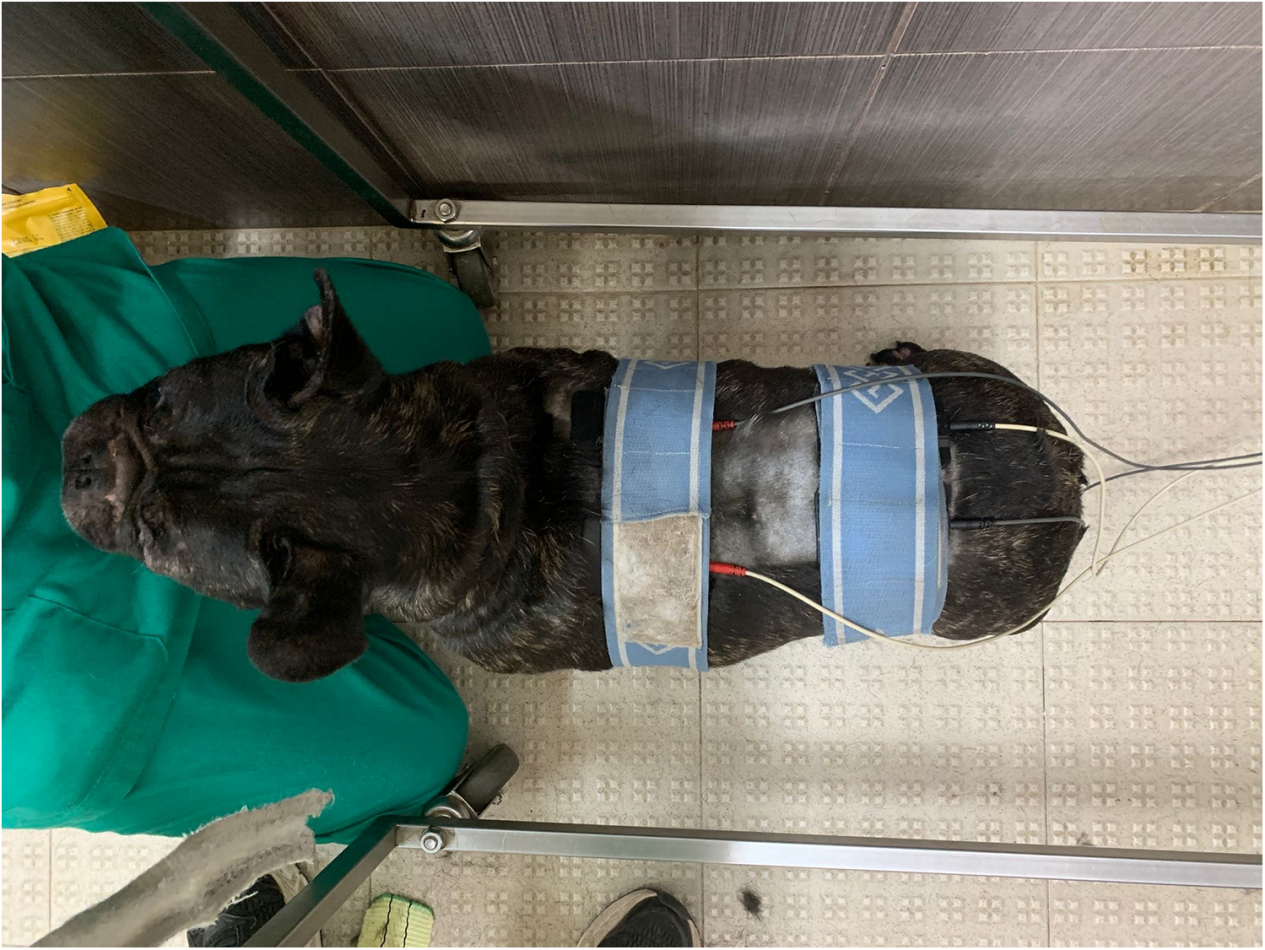
The rehabilitation modality of two-channel transcutaneous electrical nerve stimulation (TENS) with each channel placed across to the other in the paraspinal hyperesthesia region.

**Figure 7:**
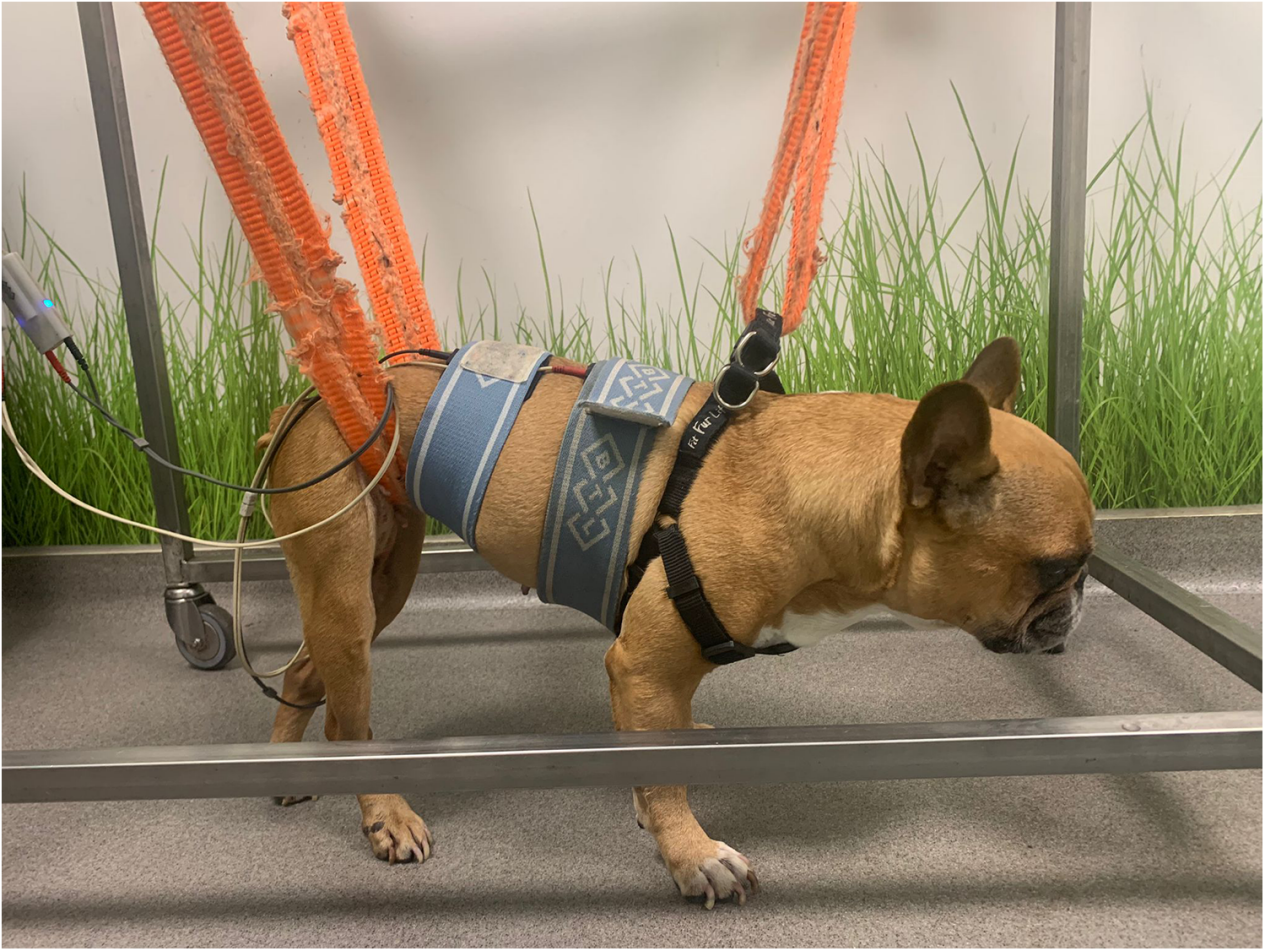
The rehabilitation modality of transcutaneous electrical nerve stimulation in a standing position.

TENS with two channels is a technique of interferential TENS based on the application of two different channels. Each channel had two superficial rubber and carbon electrodes (7 × 5 cm) placed on the paraspinal hyperesthesia region after the hair had been cut and gel had been applied. Each channel was crossed at a 90° angle, and each electrode was placed at a distance of three spinal processes cranial and caudal to the pain region (Figure 6). The device (BTL-4820 Smart, BTL, USA) was set with the following parameters, 80–150 Hz, 0.5–1 mA, pulse duration 2–50 µs, for 10 min, followed by 1–10 Hz, 0.5–1 mA, pulse duration 100–400 µs, for an additional 10 min (36, 37). The programmed current was biphasic, symmetrical, and continuous, with a limited intensity threshold to avoid muscle fasciculations or muscle contractions. The TENS was applied to all dogs in the SG once daily for 6 days/week and discontinued according to the pain assessment of the respective patient. The lack of paraspinal hyperesthesia at any given time point was deemed a criterion for not receiving TENS treatment. All dogs had to perform this technique in a standing position with the aid of a bodyweight support device (Figure 7). Thus, it was also a postural exercise to stimulate intrafusal fibers. However, If the dog felt uncomfortable during treatment, the sternal recumbency position was adopted. The results were assessed at all time points (T1–T8), in the same controlled room, with a CCRP veterinary nurse who performed the treatment, the veterinarian who did the assessments, and another veterinary nurse who made the recordings, always 5 min before the TENS for the SG.

### 2.5 Statistical analysis

All data were collected using Microsoft Office Excel 365 (Microsoft Corporation, Redmond, WA, USA), and IBM SPSS Statistics 25 software (International Business Machines Corporation, Armonk, NY, USA) was used to process all results. For the continuous variables age and weight, the Kolmogorov–Smirnov normality test (for *n* > 50), arithmetic mean, minimum, maximum, standard deviation (SD), variance, and standard error of the mean (SEM) were recorded. Arithmetic means and SD were also recorded for treatment time. Descriptive statistics with frequency analysis were generated for all categorical nominal variables, and valid percentages (percentage of sample in each category) were calculated. Chi-square tests were also performed to check for relevant analogies as evidenced by a *p* < 0.05.

## 3 Results

**Figure 8:**
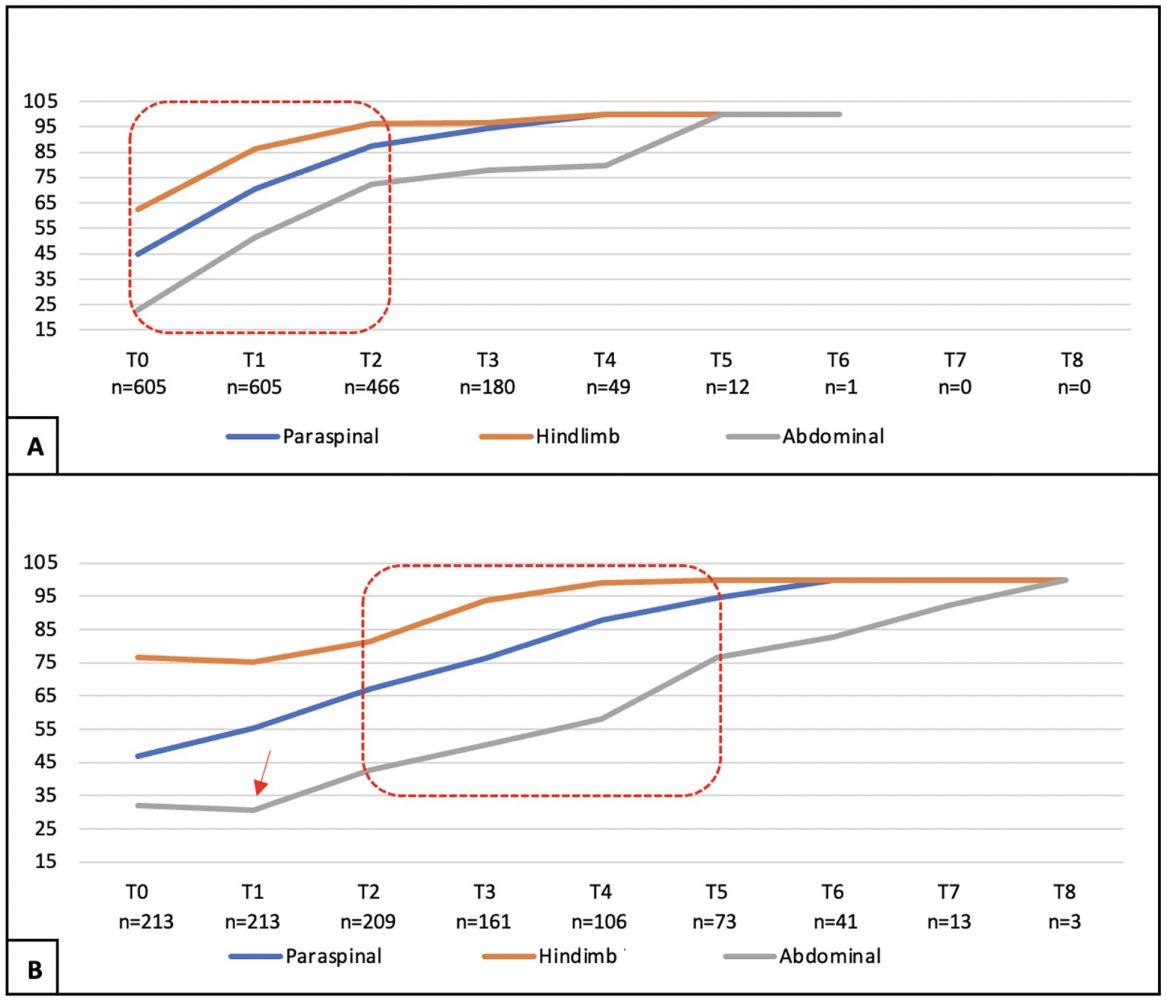
Muscle tone evaluation (paraspinal hindlimbs/abdominal) for the study group **(A)** and control group **(B)** during time points. Red marks: time of marked improvement. Red arrow: worsened abdominal tone. *X*-axis: time point T0 (admission); T1 (24 h); T2 (48 h); T3 (72 h); T4 (4 days); T5 (5 days); T6 (6 days); T7 (7 days); T8 (8 days). *Y*-axis: Percentage of dogs with normal tone.

**Figure 9:**
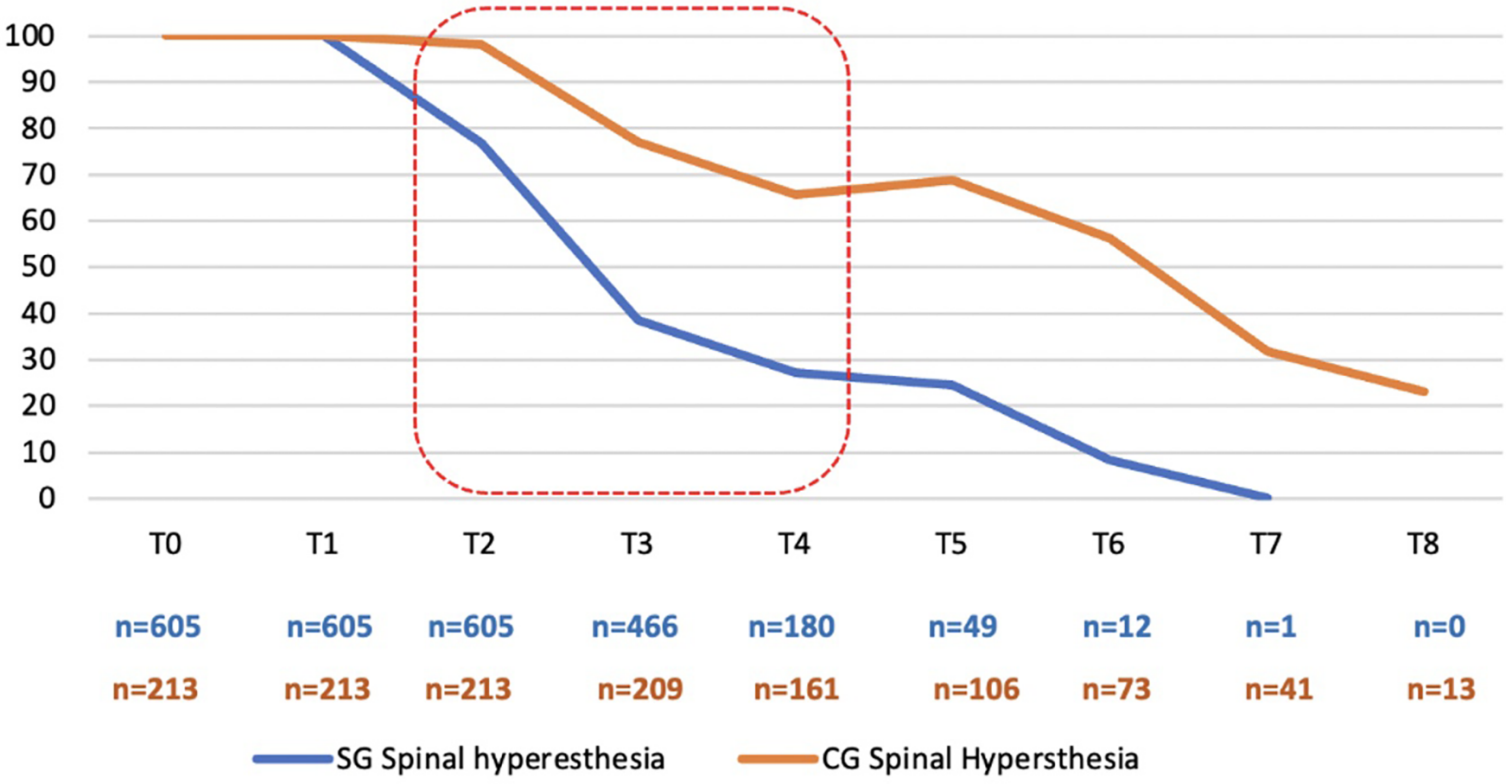
Spinal hyperesthesia evaluation during the time points. SG, study group; CG, control group. Red mark: Time of marked difference between groups. *X*-axis: Time point T0 (admission); T1 (24 h); T2 (48 h); T3 (72 h); T4 (4 days); T5 (5 days); T6 (6 days); T7 (7 days); T8 (8 days). *Y*-axis: Percentage of dogs with spinal hyperesthesia.

**Figure 10:**
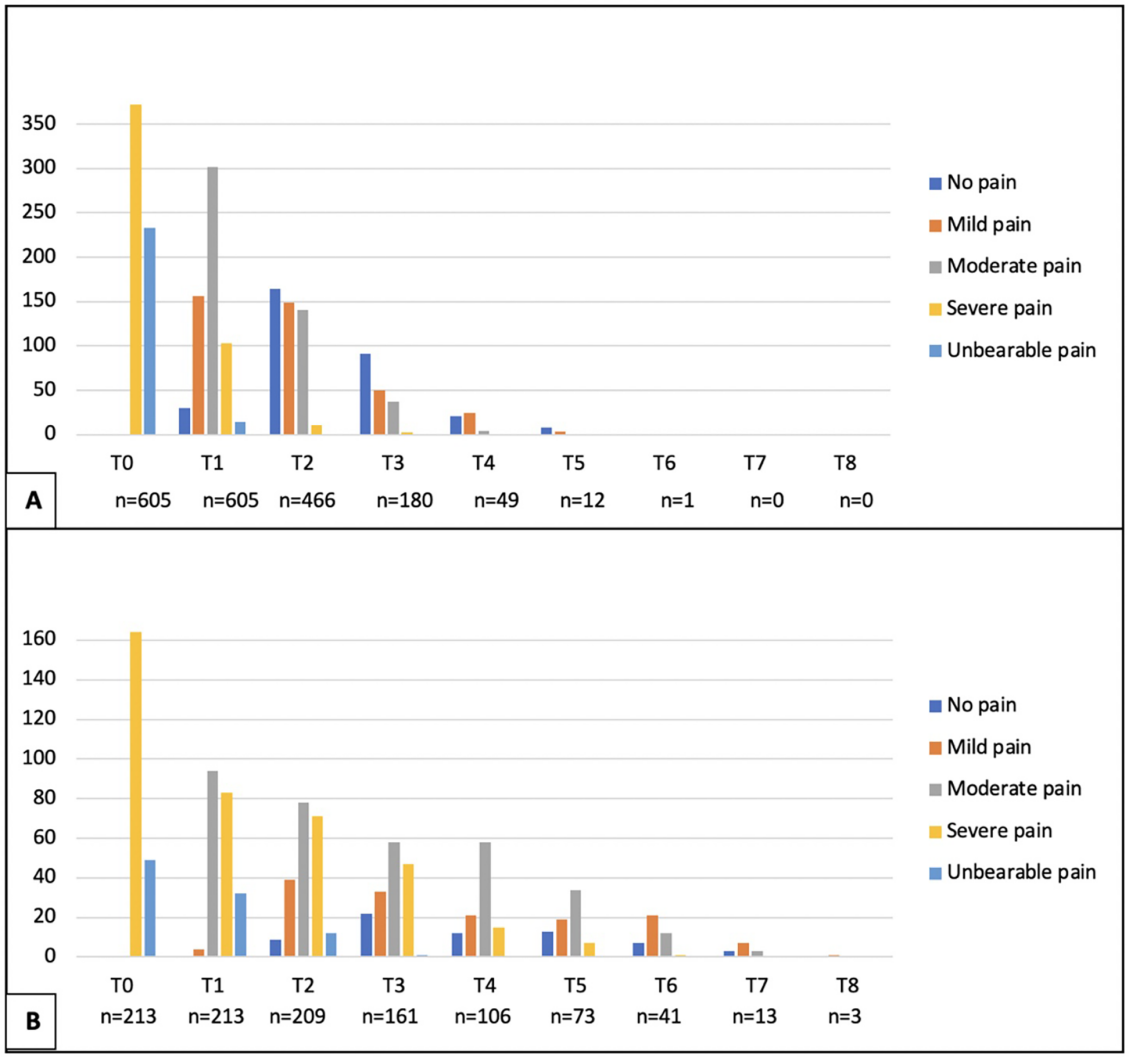
Dynamic interactive visual analog scale (DIVAS) evaluation for study group **(A)** and control group **(B)** during time points. *X*-axis: Classification of pain according to DIVAS at each time point: T0 (admission); T1 (24 h); T2 (48 h); T3 (72 h); T4 (4 days); T5 (5 days); T6 (6 days); T7 (7 days); T8 (8 days). *Y*-axis: Frequency of dogs.

**Figure 11:**
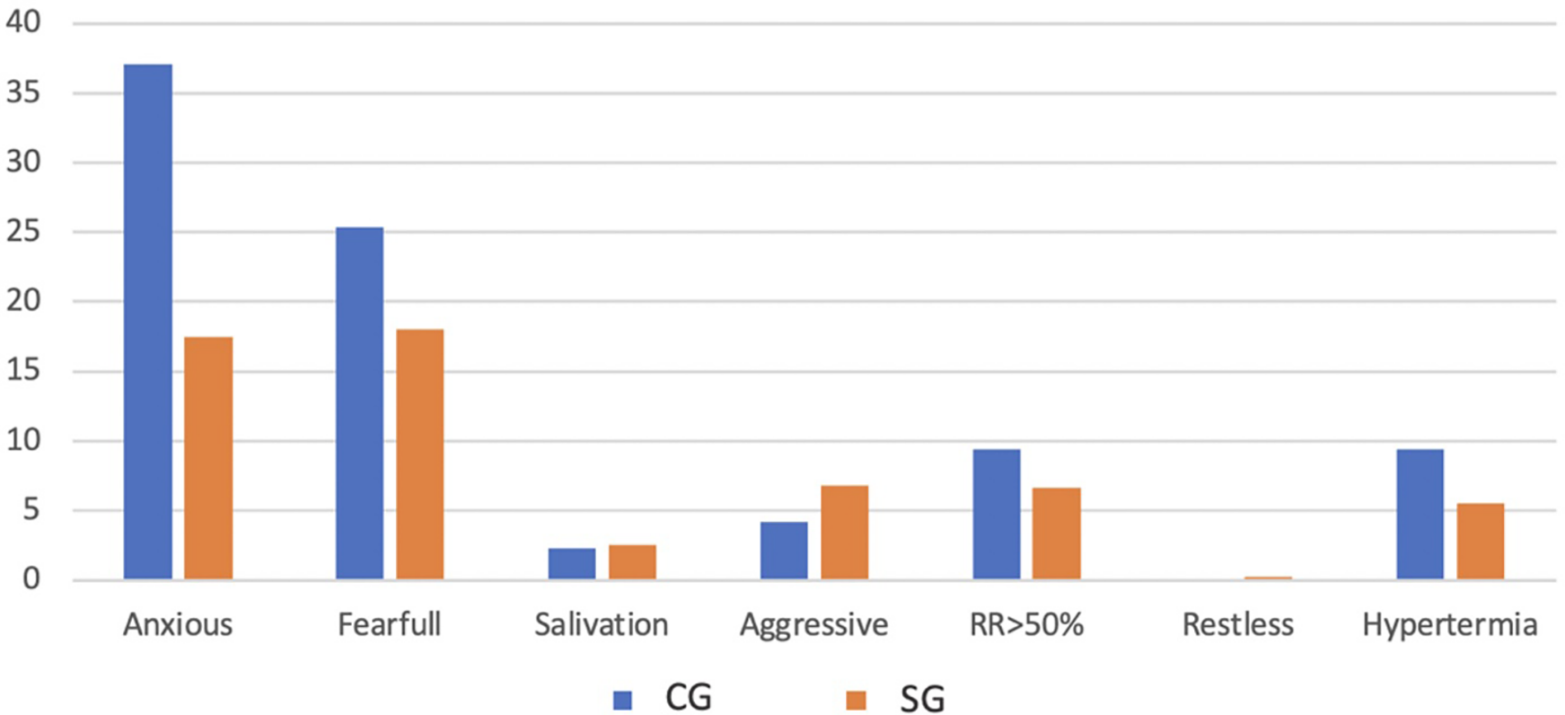
Clinical signs presented by the participants at medical discharge based on the dynamic interactive visual analog scale, considering both groups. RR, respiratory rate; CG, control group; SG, study group; *X*-axis, clinical signs presented by the dogs; *Y*-axis, percentage of dogs.

**Figure 12:**
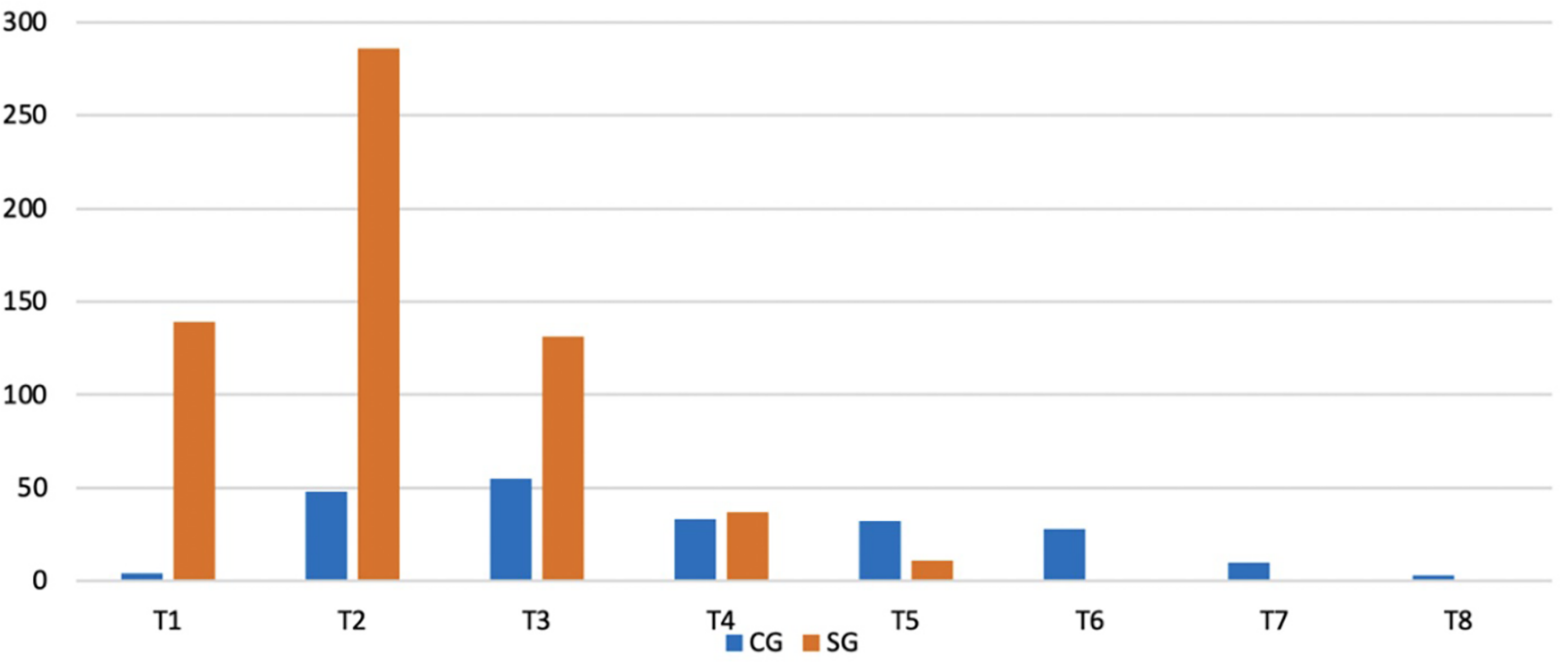
Medical discharge for dogs in both groups during time points. CG, control group; SG, study group; *X*-axis, time points: T0 (admission); T1 (24 h); T2 (48 h); T3 (72 h); T4 (4 days); T5 (5 days); T6 (6 days); T7 (7 days); T8 (8 days). *Y*-axis, frequency of dogs.

The total population was 818 dogs, allowing an approximate significance (1 − β) of 0.99 and an α (type I error) of 0.001. For the SG (*n* = 605), an approximate significance (1 − β) of 0.90 and an α (type I error) of 0.001, and for the CG (*n* = 213), an approximate significance (1 − β) of 0.50 and an α (type I error) of 0.01 was possible (38). Of the 818 dogs enrolled in this study, 60.1% (492/818) were male, and 39.9% (326/818) were female, with a mean age of 3.78 + 1.50 years (SD 1.50) and a mean weight of 11.64 kg (SD 4.84) (Table 2). The 818 dogs had the same inclusion criteria and were subdivided according to owner consent for TENS treatment. Thus, 605 dogs belonged to the SG group and underwent PSP and TENS, while 213 dogs belonged to the CG group and underwent PSP only. The mean, median, and mode for both age and weight across all groups exhibited considerable similarity; however, there was notable variability in these parameters. The Kolmogorov–Smirnov normality test (*n* > 50) indicated a significant deviation from normality (*p* < 0.001). The study comprised dogs exclusively from chondrodystrophic breeds, with the French bulldog (*n* = 348) being the most common, followed by the dachshund (*n* = 174), beagle (*n* = 129), Jack Russell (*n* = 37), basset hound (*n* = 35), shih tzu (*n* = 33), English bulldog (*n* = 25), Pekingese (*n* = 17), Boston terrier (*n* = 15), and pug (*n* = 5). The assessment of the records by the two blinded observers revealed an 18% discrepancy. During the assessments, the SG dogs demonstrated a faster recovery within the initial 48 h (T2) when compared with the CG, regarding the muscle tone of the hindlimbs, as well as in paraspinal and abdominal tone (*X*^2^(1,675) = 40.865, *p* < 0.001; *X*^2^(1,675) = 38.897, *p* < 0.001; *X*^2^(1,675) = 55.681, *p* < 0.001). The CG displayed a notable improvement between T2 and T5, with the most significant decline in abdominal tone recorded during the first 24 h (T1), as illustrated in Figure 8. All dogs exhibited paraspinal hyperesthesia upon admission (T0), and the assessment of this condition at various time points is illustrated in Figure 9. The SG group demonstrated an earlier improvement in the absence of paraspinal hyperesthesia compared to the CG group, specifically between T2 and T4 (48 h–4 days) (Figure 9). At the time of admission (T0), the SG group exhibited 61.5% (372 out of 605) of dogs experiencing severe pain, while 38.5% (233 out of 605) were suffering from unbearable pain. Conversely, the CG group presented 77% (164 out of 213) of dogs with severe pain and 23% (49 out of 213) with unbearable pain. The evolution of DIVAS for each time point in both groups is illustrated in Figure 10. At T2, only 2.4% (11 out of 466) of dogs in the study group (SG) experienced severe pain, while 34% (71 out of 209) in the control group (CG) continued to suffer from severe pain. Additionally, at this time point, 0.2% (1 out of 466) of dogs in the SG reported unbearable pain, in contrast to 5.7% (12 out of 209) in the CG. By T3, only 2 dogs in the SG still exhibited severe pain, compared to 47 dogs in the CG. Throughout the duration of the study, instances of severe pain in the CG were observed to persist until T6 (refer to Figure 10). From T0 to T1, the SG (*n* = 605) demonstrated a remarkable improvement of 99.8% (604 out of 605) in their scores, with only 0.2% (1 dog) showing a decline. In contrast, the CG (*n* = 213) had 86.4% (184 out of 213) of dogs improving, while 9.4% (20 out of 213) experienced a decline, and 4.2% (9 out of 213) maintained the same score. The difference in the DIVAS score between the study and control groups was statistically significant [*X*^2^(2,818) = 80.736, *p* < 0.001]. From T1 to T2, the SG (*n* = 466) showed a 99.1% (462 out of 466) improvement, whereas the CG (*n* = 209) had a 94.7% (198 out of 209) improvement. Notably, no dogs in the SG experienced a decline, while the CG had one dog that got worse. Regarding DIVAS, a significant number of dogs exhibited signs of anxiety and fear upon medical discharge. Specifically, 17.5% (106 out of 605) of the dogs in the SG were identified as anxious, while 18% (109 out of 605) displayed fearful behaviors. In contrast, the CG showed higher percentages, with 37.1% (79 out of 213) of dogs experiencing anxiety and 25.4% (54 out of 213) exhibiting fear (see Figure 11). A significant number of the dogs exhibiting a DIVAS with a respiratory rate greater than 50, along with signs of fear and hyperthermia, were French bulldogs, with 20 in the CG and 26 in the SG. In terms of aggressive behavior, the majority were beagles, comprising 4 in the CG and 15 in the SG. Paraspinal hyperesthesia was no longer observed at the time of discharge, which was sooner for the SG compared to the CG, with a significant difference between groups [*X*^2^(7,818) = 245.165, *p* < 0.001]. The SG had an average discharge time of 2.17 days (SD 0.924), while the CG had an average of 3.94 days (SD 2.037). In terms of medical discharge, it was noted that three dogs in the CG remained hospitalized until T8, whereas T6 was the latest discharge duration in the SG, and was only observed in one SG dog (Figure 12). Also, there were no adverse effects detected during treatments. In the follow-up analysis, it was observed that 82% (496 out of 605) of the dogs from the SG attended the assessment F1, whereas only 43% (213 out of 496) returned for F2. In the CG, 78% (166 out of 213) of the dogs were present in F1, with a subsequent attendance of 70% (116 out of 166) in F2. Notably, there were no indications of paraspinal hyperesthesia in either group during the follow-up assessments.

**Table 2:** Descriptive analysis of age and weight in the total study population, study group, and control group.

Descriptive analysis parameters (age and weight)		Total study population (*n* = 818)	SG (*n* = 605)	CG (*n* = 213)
Age	Mean	3.78	3.79	3.76
	Median	4	4	4
	Mode	3	3	3
	Variance	2.243	2.346	1.957
	SD	1.498	1.532	1.399
	Minimum	1	1	1
	Maximum	7	7	7
	SEM	0.052	0.062	0.096
	Kolmogorov–Smirnov normality test	*p* < 0.001		
Weight	Mean	11.64	11.54	11.94
	Median	11	10	11
	Mode	11	9	11
	Variance	23.387	24.024	21.567
	SD	4.836	4.901	4.644
	Minimum	2	2	3
	Maximum	25	25	24
	SEM	0.169	0.199	0.318
	Kolmogorov–Smirnov normality test	*p* < 0.001		

SD, standard deviation; SEM, standard error of the mean; SG, study group; CG, control group.

## 4 Discussion

This clinical study represents the efficacy of TENS in veterinary medicine using two channels as a neuro-modality for dogs suffering from acute paraspinal thoracolumbar acute hyperesthesia, without surgical intervention. The dogs involved in this study were assessed using the dynamic interactive visual analog scale, with a recorded DIVAS grade of 14 or higher. The application of TENS was a supplementary treatment alongside the standard pharmacological therapy, mirroring human medicine. As a matter of fact, these treatments can be translational for other animal species (1, 3).

A total of 818 dogs were categorized into the SG and CG groups, exhibiting comparable median values, as well as median and mode statistics concerning age and weight (see Table 2). Furthermore, all dogs of a chondrodystrophic breed likely demonstrated the expression of the fibroblast growth factor 4 (FGF4) retrogene located on chromosome 12, which is linked to accelerated degeneration of intervertebral discs (21, 39–42). It is essential to acknowledge the variation in group sizes, despite the implementation of stringent inclusion criteria, as this may influence the interpretation of results. Furthermore, the application of subjective outcome measures, such as DIVAS and the outcomes checklist, may introduce potential measurement bias, which could account for the 18% discrepancy observed between blinded observers. More objective tools (i.e., pressure algometer or von Frey filaments) could be used for further studies (43–45). Conversely, the selection of DIVAS for assessing paraspinal hyperesthesia was driven by the requirement for both visual and palpation assessments. Consequently, in the absence of a validated scale in veterinary medicine for evaluating complex or mixed acute pain in dogs undergoing conservative treatment, the palpation technique remains the predominant method employed (31). The groups were also similar at admission, in terms of the normal response to the palpation of muscle tone, despite the absence of validated measurement tools and the inherent physiological variability among individuals. Additionally, variability may be also related to skin conductivity when using TENS, due to individual differences in electrical conductance controlled by the sympathetic and parasympathetic nervous system, leading to different tolerability thresholds. However, by standardizing the parameters of frequency, location, and distance of the superficial electrode, efforts can be made to minimize potential bias. An assessment of the dog's response to paraspinal, limb, and abdominal muscle palpation was conducted. A significant proportion of dogs exhibiting acute severe to intolerable pain demonstrated a high prevalence of increased muscle tone, with 77.2% showing elevated abdominal tone in the SG, necessitating assistance from the PSP. Benzodiazepines, including diazepam and midazolam, which are frequently utilized in clinical practice, were excluded from this study to prevent any potential confusion concerning the outcomes associated with the two-channel TENS.

This outcome assessment regarding muscle tone, although not yet validated, indicated a more rapid improvement in the SG during the initial 48 h (from T0 to T2). Additionally, a significant enhancement was observed from T2 to T5 (the second to the fifth day), in contrast to a noticeable delay in recovery for the CG, as illustrated in Figure 8. This difference may be attributed to the effective alleviation of mixed pain through the application of transcutaneous electrical nerve stimulation (TENS). Research involving human patients with fibromyalgia has demonstrated that TENS can provide effective analgesia (7, 46–50), primarily through the use of low-voltage electrical impulses delivered via superficial electrodes on the skin, employing various waveforms, frequencies, and amplitudes. Conventional TENS (≥50 Hz) activates the large cutaneous A*β* fibers, which inhibit neurons (48, 51), thereby an antidromically current may suppress the nociceptive signals entering the dorsal horn. Paraspinal hyperesthesia has been identified as a combination of nociceptive, inflammatory, and neuropathic pain (74). This condition is characterized by an increased sensitivity to stimuli, exhibiting various manifestations that were examined by the DIVAS across our entire canine population, beginning at T0. At this initial assessment, all dogs exhibited severe pain (61.5% in the SG and 77% in the CG) or unbearable pain (38.5% in the SG and 23% in the CG). Consequently, the pharmacological standard protocol (PSP) implemented for all dogs was centered around gabapentin, a widely utilized anticonvulsant for managing neuropathic pain, typically administered at a dosage of 10–20 mg/kg TID (52–54). However, due to the multidisciplinary nature of the protocol, the dosage prescribed in this study was adjusted to 5 mg/kg TID, in conjunction with non-steroidal anti-inflammatory drugs (NSAIDs), paracetamol, and opioids (methadone). Implementation of the PSP was justified by the signs of paraspinal hyperesthesia, which in humans, is manifested as sensations such as burning, shooting, pricking, tingling, squeezing, or freezing pain (55, 56). These sensations, characterized by tingling and pulsating pain, arise from the activation of large A*δ* and A*β* fibers (57, 58), as well as ectopic nerve impulses in large, rapidly conducting myelinated mechanoreceptive fibers. Additionally, burning pain may be triggered by the stimulation of C nociceptive fibers through interneural pathways (59). The mechanism by which pain impulses are blocked from being transmitted to the brain and subsequently controlled was articulated by Melzack and Wall in 1965. It was established that the activation of the “gate control” theory resulted from antidromic stimulation. Consequently, it was suggested that this activation of gate control could influence pain perception through the engagement of certain supraspinal pain processing systems, including the “spino-bulbo-spinal loop” (60). The SG exhibited a more rapid and earlier improvement. On the fourth day (T4), 70% (*n* = 425) of the dogs in the SG showed no signs of pain, whereas only 24% (*n* = 52) in the CG were pain-free. By the sixth day (T6), the SG reached a medical discharge rate of 98% among all dogs (*n* = 593), in contrast to the CG, which achieved a discharge rate of only 66% (*n* = 140). In examining the evolution of the DIVAS at various time points, both groups experienced a reduction in pain scores, indicating that the PSP effectively alleviated mixed complex pain in this canine population. Notably, the SG exhibited the earliest signs of pain relief (Figure 10). Furthermore, the DIVAS score improved by 99.8% (*n* = 604) in the SG, with only one dog experiencing a deterioration of this score. In contrast, the CG showed an improvement rate of 86.4% (*n* = 184), although 11 dogs either worsened or maintained their pain scores (*p* < 0.001). It is essential to consider the validation status of the assessment tools employed in these evaluations.

The literature indicates that TENS facilitates the release of endogenous opioids (12, 48, 61). In the study, the two-channel TENS device was configured with both channels set to the following parameters: 80–150 Hz, 0.5–1 mA, and a pulse duration of 2–50 microseconds for a duration of 10 min. This configuration, categorized as high frequency, is predominantly utilized for managing acute pain (1, 61–63). Subsequently, both channels were adjusted to as follows: 1–10 Hz, 0.5–1 mA, with a pulse duration of 100–400 microseconds, also for 10 min (37). This low-frequency TENS is typically employed for the treatment of chronic pain (9, 11, 61, 63). This 20 min TENS treatment may address the complexity of mixed pain and the refractory nature of neuropathic pain (74). The efficacy of combining NSAIDs with gabapentin has been established regarding general neuropathic pain (64). Furthermore, when supplemented with TENS therapy, dogs showed significant improvement between T1 (24 h) and T2 (48 h), with all dogs in the SG showing improvement in their DIVAS scores, while only 11 dogs in the CG did not show similar progress. The SG dogs exhibited a reduction in perceived pain, likely attributed to the enhancement of inhibitory descending pathways involving serotonin, noradrenaline, dopamine, acetylcholine, and opioids. Concurrently, there was a decrease in the nociceptive ascending pathways, facilitated by an increase in endocannabinoids, opioids, and GABA, or by the inhibition of glutamate, P-substance, IL-1β, and IL-6 (60). TENS is commonly employed in veterinary practice for dogs and cats to achieve pain relief (33, 37). This method is favored due to its non-invasive nature, cost-effectiveness, safety, and ease of application (1, 62). In human medicine, TENS is utilized for managing various pain conditions (1, 65, 66). The arrangement of superficial electrodes in a crossing pattern at a 90° angle, traversing the area of paraspinal hyperesthesia, is anticipated to enhance spinal segmental effects, including the reduction of inflammation and modulation of the activity of supportive cells surrounding neurons, such as glial cells (7, 16). These effects are reported to inhibit the transmission of nociceptive signals at the spinal cord level (19, 67). Furthermore, the frequency parameter is crucial, with effects having been validated in human subjects (19, 68–71). Additionally, the intensity of stimulation is significant for enhancing the efficacy of the two-channel TENS treatment, which should be maintained at a maximum of 2.5 mA to ensure patient comfort (37, 72). Therefore, to effectively manage or alleviate mixed complex pain, TENS must utilize appropriate stimulation parameters that are vital for enhancing analgesic effects and improving patient well-being (17). Although no signs of discomfort behaviors were noted in the SG, there was an increase in respiratory rate (RR > 50), as well as instances of fear and hyperthermia, observed in both groups, primarily associated with the hospitalization environment. This situation may lead to the emergence of respiratory stress disorders, particularly evident in brachycephalic breeds like the French bulldog (73).

Time until discharge was different between the groups, with the SG averaging 2.17 days, while the CG had a mean of 3.94 days, indicating a duration that was twice as long (Figure 12). The study’s findings further revealed that both groups experienced pain management during F1 and F2. Nevertheless, dogs in the SG diminished to 43% compared to F1, which could pose a limitation for the assessment of long-term follow-up data. The primary limitation of this study lies in the lack of objective evaluation outcomes and the failure to validate the DIVAS and outcomes checklist. The assessment of mixed complex pain, encompassing both nociceptive and neuropathic components, relies solely on subjective measures derived from an outcome's checklist, demanding more rigorous standards for validity, reliability, and accuracy. Furthermore, future research could benefit from the incorporation of biomarkers that may provide insights into paraspinal hyperesthesia.

## 5 Conclusion

In this study, both groups demonstrated improvement over the designated time intervals; however, the SG exhibited a more rapid recovery concerning pain indicators, as evidenced by a reduction in DIVAS classification, which subsequently lessened the reliance on pharmacological interventions. Additionally, a significant decrease in the duration required for clinical discharge was observed, nearly halving the time for the group treated with TENS, despite the use of a non-validated checklist for outcome measurement. Due to the limitations of this study, further research should focus on defining specific and reliable metrics to assess pain.

## Data Availability

The raw data supporting the conclusions of this article will be made available by the authors, without undue reservation.
